# Brassicaceae Mustards: Traditional and Agronomic Uses in Australia and New Zealand

**DOI:** 10.3390/molecules23010231

**Published:** 2018-01-21

**Authors:** Mahmudur Rahman, Amina Khatun, Lei Liu, Bronwyn J. Barkla

**Affiliations:** Southern Cross Plant Science, Southern Cross University, Lismore, NSW-2480, Australia; m.rahman.21@student.scu.edu.au (M.R.); a.khatun.10@student.scu.edu.au (A.K.); ben.liu@scu.edu.au (L.L.)

**Keywords:** Brassicaceae oilseeds, bioactive constituents, canola, mustard, glucosinolates, agronomic importance, Australia and New Zealand traditional medicine

## Abstract

Commonly cultivated Brassicaceae mustards, namely garlic mustard (*Alliaria petiolata*), white mustard (*Brassica alba*), Ethiopian mustard (*B. carinata*), Asian mustard (*B. juncea*), oilseed rape (*B. napus*), black mustard (*B. nigra*), rapeseed (*B. rapa*), white ball mustard (*Calepina irregularis*), ball mustard (*Neslia paniculata*), treacle mustard (*Erysimum repandum*), hedge mustard (*Sisymbrium officinale*), Asian hedge mustard (*S. orientale*), smooth mustard (*S. erysimoides*) and canola are the major economically important oilseed crops in many countries. Mustards were naturalized to Australia and New Zealand and Australia is currently the second largest exporter of Brassicaceae oilseeds to meet the global demand for a healthy plant-derived oil, high in polyunsaturated fats. Apart from providing edible oil, various parts of these plants and many of their phytochemicals have been used traditionally for both agronomic as well as medicinal purposes, with evidence of their use by early Australian and New Zealand settlers and also the indigenous population. This review provides an overview of the current knowledge of traditional and agronomic uses of Brassicaceae oilseeds and mustards with a focus on their importance in Australia and New Zealand.

## 1. Introduction

Brassicaceae comprise a diverse family of plants and provide one of the most extensive and varied range of end products used by man from a single plant genus. Mustards are members of the Brassicaceae family, and are among the earliest cultivated plants. Their seeds are one of the oldest recorded spices with use and cultivation dating back over 5000 years [1,2,3].

**Figure 1:**
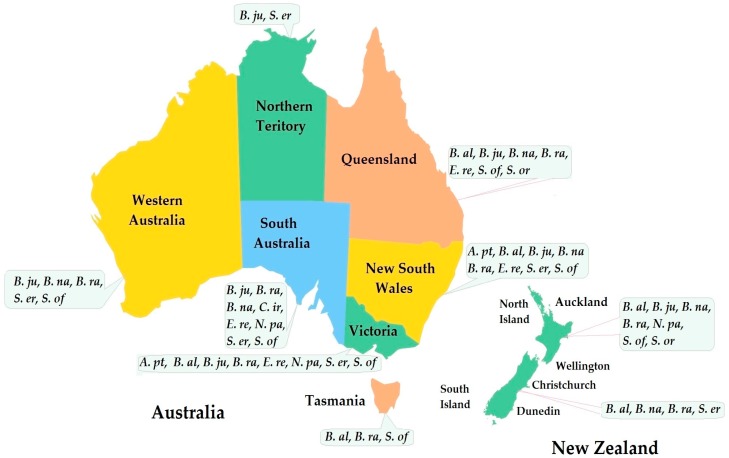
Distribution of mustards in Australia and New Zealand. *A. pe* = *Alliaria petiolata*, *B. al* = *Brassica alba*, *B. ju* = *B. juncea*, *B. na* = *B. napus*, *B. ni* = *B. nigra*, *B. ra* = *B. rapa*, *C. ir* = *Calepina irregularis*, *E. re* = *Erysimum repandum*, *N. pa* = *Neslia paniculata*, *S. of* = *Sisymbrium officinale*, *S. or* = *S. orientale* and *S. er* = *S. erysimoides*. Box represents in which state or region the species are distributed. The figure is adapted from [16,17,18,19], Atlas of Living Australia, Australia’s Virtual Herbarium, Flora of Australia Online, Flora of New Zealand, Plants for the Future Database, National Library of New Zealand, New Zealand Plant Conservation Network, Auckland Museum (http://www.aucklandmuseum.com/collection/object/) and http://www.herbiguide.com.au.

Literature suggests that, within the history of human settlement in Australia and New Zealand, different types of Brassicaceae mustards, namely *Alliaria petiolata*, *Brassica alba*, *B. carinata*, *B. juncea*, *B. napus*, *B. nigra*, *B. rapa*, *Calepina irregularis*, *Erysimum repandum*, *Neslia paniculata*, *Sisymbrium officinale*, *S. orientale* and *S. erysimoides* have been naturalized (Figure 1), and adapted for use as food, incorporated into traditional medicine and play an important role in the agriculture of the two countries [4,5,6,7,8,9,10,11,12,13,14,15].

Mustards have been consumed for centuries as vegetables, and their products used as condiments and as edible and industrial oils [2,20]. The oil is commonly used for cooking and to add a hot and spicy flavor to food [7]. As a crop, they are also one of the highest oil yielding and high protein containing oilseed species. Economically important members of this family include vegetables like broccoli, cabbage, Chinese cabbage, turnip, and cauliflower, and the seed oil crop canola [18,21]. Canola is the second most economically important edible oilseed crop worldwide, with annual world production in excess of 73 million tons. It is the third-most important crop to Australian agriculture after wheat and barley, and second most cultivated oilseed after soybean, with an annual production of about 3.5 million tons [2,22]. According to the crop reports of Australian Oilseeds Federation and first quarter report of Agricultural commodities by Australian Bureau of Agricultural and Resource Economics and Sciences in 2017, less than 20% of the rapeseed (largely canola) oil produced in Australia stays in the country. Over 80% of this commodity is exported to Europe of which almost all is directed to bio-fuel production [23]. Australia supplies about 20% of the global demand for edible canola oil [24]. The edible oil contains low pungency, low erucic acid (less than 2%) and low aliphatic glucosinolate (less than 30 µmol/g). These low levels have been obtained from designed breeding of several cultivars of *B. juncea*, *B. napus*, *B. rapa* and *S. alba* [25,26]. Australian canola grade oilseed crops consistently show on average a 2% higher oil yield than worldwide averages (41.8% compared to 40% oil per dry seed weight) [27] and yields more oil per unit of land area compared to any other oilseed crop [21,28]. Canola oil is one of the most popular edible and healthy cooking oils due to its low saturated fatty acid content, high mono-unsaturated fatty acids, and balanced content of omega-3 fatty acids. Canola oil is also used to manufacture condiments, as a preservative, and as taste and flavor enhancers in pickles, chutney and other processed foods [29]. Australia exports the surplus seed meal after oil extraction as a source of animal protein [30]. Canada is the largest producer and exporter of canola grade rapeseed which mainly grows genetically modified canola (GM) whereas the canola grade rapeseed grown in Australia is non-GM [23].

Like Australia, oilseed rape is the most important oilseed crop in New Zealand where it is the most popular food grade cooking oil and used to manufacture spreads, dressings and for biofuel production. The oil content is similar to the global average at about 40%. According to the United States Department of Agriculture statistics, about 55,000 metric tons of rapeseed oil was consumed in New Zealand in 2017. The meal is used commonly as a protein source for the livestock and aquaculture industries. New Zealand harvests approximately six thousand tons of Brassica seed per year, two thirds of which are exported [31,32,33]. In New Zealand, rapeseeds are mainly cultivated in Oamaru, North Otago. The world record of canola yield was set at 6.5 tons per hectare in 2015 in that region [34].

The occurrence of various mustard species in the flora of New Zealand has been documented, with high plant numbers documented for the Canterbury Plains and Otago, Alpine wetlands and grasslands and in the Mount Cook region [35,36]. It is believed that mustards were brought to New Zealand by the British explorer James Cook and navigator Tobias Furneaux in the seventeenth century and over time several natural hybridizations occurred among them [36]. The indigenous Māori, also known as Te Reo, have established a unique culture known as “Māori Rongoā Māori”- the traditional healing system, which holds the belief that several plants are the spiritual causes of illness and therefore considered as essential for healing. They have a wide range of beliefs on the beneficial and prejudicial healing effects of mustards and they used mustard as spices and for medicinal purposes [31,37], including to treat gastric irritation and puerperal convulsions [38,39,40].

Oceania, comprising the countries of New Guinea, Micronesia, Melanesia, Polynesia, in addition to Australia, and New Zealand, has a rich traditional medicinal system that has developed from the knowledge, practices and lore of early settlers in this area, and has now been passed through generations. Evidence of the use of mustards is reported in the early literature of this area [37,38,39,40,41].

*Alliaria petiolata* (M.Bieb.) Cavara & Grande (synonym-*Alliaire officinalis, Sisymbrium alliaceum, S. alliaria*), commonly known as garlic mustard is an invasive, biennial herb native to a number of countries in temperate and tropical Asia, Africa, North America and Europe. In Australia, it is naturalized in New South Wales (NSW) and can be found in the Macedon ranges in Victoria. In New Zealand, it is documented around Ashburton, Canterbury, and Westland regions near Christchurch [14,17,42,43]. Young tender leaves of this plant have a special pungent garlic-like smell giving the plant its common name [44] and are commonly chopped to add to salad. It is used as an antiseptic in ulcers and cuts, as disinfectant, a diuretic and to heal wounds and bronchial complications [45]. Leaves of this plant have more vitamin A than spinach leaves and more vitamin C than oranges [46,47]. It is listed in the “Substances that May be Used in Listed Medicines in Australia” by Therapeutic Goods Australia (TGA), the medicinal product regulatory body in Australia, for its medicinal properties [48].

*Brassica alba* Boiss. (Hook f. & Th.), synonym-*B. hirta* Moench., *Sinapis alba* Linn, commonly called white mustard or yellow mustard, is the best known mustard in Europe, first used around 400 BC. It is the hottest known flavor for use in the Mediterranean region and was carried by explorers and cultivated in India and China due to its popularity [14,15]. It has been used in Australia since the early European settlement for culinary and medicinal purposes [44]. White mustard is a fast growing salad crop [49]. The seedling leaves have high vitamin A, C and E content and, in medicinal medicine are used to purify and strengthen the blood [14,44,49]. The seeds have strong disinfectant properties and are used to preserve foods [15,49]. For the same reason, an infusion in boiling water is used in Australia as a gargle for cold, cough and treatment of sore throats [44].

*Brassica carinata* A. Braun., known as Ethiopian or Abyssinian mustard, is a native traditional African vegetable cultivated in the Ethiopian highlands [50,51]. It is thought to be an amphidiploid between *B. nigra* and *B. oleracea*. It would usually be spring sown for seed crops in Canterbury, New Zealand. The plant is widely used as bio-fumigant, to suppress soil-borne pests and pathogens and because of this is used as a rotation crop by farmers in Australia and New Zealand [52].

*Brassica juncea* (L.) Czerniak. Coss., synonym-*B. integrifolia*, is variously known as brown mustard, Asian mustard, Oriental mustard, Chinese mustard, Indian mustard, leaf mustard, giant red, sarepta mustard, Asiatic mustard, mustard green, and wild Brazilian mustard. It is an annual herb native to eastern and southern Asia. It is widely cultivated throughout India, central Africa, and southern Russia, and Caspian steppes. The variety of *B. juncea* cultivated in Australia is a hybrid one compared to that of native Asian mustard [53], which is a natural hybrid between *B. rapa* and *B. nigra* [53,54,55,56,57,58]. *B. juncea* is also listed in the Australia and New Zealand Food Standards Code (Schedule 22—Foods and classes of foods). The potential of *B. juncea* to adapt to the low rainfall environment of areas of Australia was recognized in the early 1980’s and a canola quality *B. juncea* was developed, with low erucic acid, moderate oleic acid and low glucosinolate levels [59,60]. The leaves are used as salad and Australians, and New Zealanders use the seed oil as an edible oil instead of olive oil [59]. *B. juncea* is also used in Australia and New Zealand as fodder and in traditional medicine as anodyne, aperitif, diuretic, emetic, rubefacient, and stimulant. Herbal medicinal practitioners use it to treat arthritis, foot-ache, lumbago, and rheumatism [53,61]. The seeds are high in vitamin A and K and when eaten uncooked, the oil is ‘nose clearing’ [55]. The flowering shoots are tremendously hot when eaten fresh, with a peppery taste, but mild when cooked [49]. While investigating the traditional Australian Aboriginal and Indian Ayurvedic medicinal plants for their role in management of type 2 diabetes, the leaves of *B. juncea* were found to decrease hyperglycemia [62].

*Brassica napus* (var. napus or var. oleifera), synonym-*B. rutabaga*, vernacularly named as rapeseed, oilseed rape, cole, colza, rutabaga, swede, Swedish turnip, rape, leaf rape, raps, and rapsfromel, is a winter oilseed rape, and a summer rape. Native to south-east Asia and Eurasia, it is most commonly grown in northern temperate regions including northern Asia, Japan, Korea, Northern China, Scandinavia and Russia [63,64,65]. In Australia, *B. napus* has been naturalized in Western Australia, New South Wales, Queensland, and Victoria and to the Southern Island regions of Marlborough and Canterbury in New Zealand [66]. Napus (rape or coleseed) and naprobrassica (L) Reichb are the two most cultivated varieties in Australia and mostly used to produce oils and as fodder and some medicinal purposes [53]. In Australia and New Zealand *B. napus* is used in stews, soups and as a flavor enhancer [44].

*Brassica nigra* (L.) W.D.J. Koch. synonym- *Sinapis nigra*, *B. sinapioides*, commonly known as black mustard, brown mustard, moutarde noire and senf. is an annual herb native to most parts of Europe, the Mediterranean region, and other parts of north Africa and has been naturalized in Great Britain and North America, and in the Canterbury and Otago regions of New Zealand [66]. Use of the active constituent, allyl isocyanate, of *B. nigra* and spp., either internally or externally, is not recommended for therapeutic purposes in Australia due to the irritant effect of the chemical [67]. In Australia, black mustard oil is utilized for production of soap and for medicinal remedies; the seeds are valued as a stimulant, irritant, emetic and to treat bronchitis [44,53]. Regular consumption of black mustard seeds is reported to improve the body’s biological defense mechanisms against cancer development and studies have shown that it can reduce the rate of colon, bladder and lung carcinogenesis [55]. It relieves congestion by drawing the blood to the surface as in head afflictions, neuralgia and headaches [55]. In another Australian study it was suggested that the antioxidant activity and polyphenolic content of *B. nigra* can inhibit pancreatic α- amylase enzyme resulting in improved glucose tolerance in diabetics [62]. The α-amylase enzyme is one of the key enzymes in the small intestine for the digestion of starch, breaking it down to glucose and maltose which leads to increased postprandial glucose levels and inhibition of this enzyme is one of the pathways to manage diabetes [68].

*Brassica rapa* L., synonym- *B. campestris* L., var. *sylvestris* (Lam.) Briggs [53], well known as oilseed rape, rape oilseed, kale rape, rapa, rappi, keblock, colza, bird rape, rape mustard, field mustard, wild mustard, turnip mustard or rapeseed and in the case of one particular group of cultivars, canola, is the most cultivated *Brassica* oilseed in many countries including Australia and New Zealand [69,70]. It is native to central Asia and Europe, naturalized in New South Wales, Queensland, Western Australia, southern Victoria and Tasmania in Australia; as well as the South Island in New Zealand [41,57,71]. This annual or biennial winter crop grows wild in Central Otago, and is known as “paea, pōhata and pōwhata” among the Māori people who eat the large white roots and use the leaves of the plant medicinally [40,41]. All parts of this species, including the leaves, stems, seeds and roots are utilized for food [65]. The seeds are considered highly nutritious because of the high content of vitamin A, B_1_, B_2_, and C, as well as protein with a balanced amino acid profile [59]. In Australian herbal medicine, its use has been documented as a liniment to relieve aching muscles, disinfect wounds, and in plasters for the remedy of chest congestion and bronchitis [72,73].

*Calepina irregularis* (Asso.) Thellung. known as white ball mustard, smooth ball mustard, and mustard spinach is native to countries in central Asia, and the Mediterranean basin and naturalized in central Europe North America and Australia [74,75]. Introduced species are recorded in South Australia, and Victoria ([76], Flora of Australia Online), but it is not documented in New Zealand. In Australia, it is sometimes cultivated as a rotational crop. The leaves are eaten as salad or boiled in curries [77].

*Erysimum repandum* L., synonym *E. cheiranthoides* L., *E. perfoliatum, E. orientale* and *Conringia orientalis* (L.) C. Presl., known as treacle mustard and hare’s-ear mustard, is a common weed in Australia and New Zealand. This weed originated from the Mediterranean, southern Europe and North America [53] and was naturalized in Australia and New Zealand [53,66,78]. Treacle mustard is distributed in New South Wales, Queensland, South Australia, and Victoria in Australia [77]; as well as Balclutha and Waitepeka in New Zealand [53,66,78]. The leaves can be eaten as a vegetable [77].

*Neslia paniculata* (L.) Desv., synonym *Myagrum paniculatum*, vernacularly known as ball mustard, common ball mustard, yellow weed, neslia, neslie or moutarde is a native Euro-Siberian southern-temperate species. It is naturalized in Europe, Asia, Canada and South America. In Australia, it is found in north-west Victoria, and South Australia; while in New Zealand it is found near Palmerston North [14,76]. Apart from the culinary uses, the plant is used by folklore medicinal practitioners for its curative value [53].

*Sisymbrium officinale* L. Scop., synonym *Erysimum officinale*, known as erysimum, English watercress, hedge mustard, St. Barbara’s hedge mustard, common hedge, singer’s plant, and thalictroc, is an annual or biennial mustard. It is found on roadsides, wastelands and as a weed of arable land in Eurasia, the Mediterranean, north-western Africa, Scandinavia and Asia, and naturalized in Australia and New Zealand [79,80,81]. Hedge mustard is sometimes regarded as an environmental weed in the Australian Capital Territory, Victoria and South Australia [82]. It has been listed for official medicinal plant use in Australia. In Australian and New Zealand folk medicine, the seed and plant extracts were made into a syrup, with honey or sugar and flowers, and used to make a strong infusion. The whole plant, which is highly pungent is often infused for treatment of sore throat and as an expectorant to treat common cold and asthma where the chest is highly congested. It gets its name ‘singer’s plant’ for these cures [83].

*Sisymbrium orientale* L., synonym *S. columnae* Jacq., known as Asian hedge mustard, Indian hedge mustard, eastern rocket, oriental wild rocket and London rocket, is native to Europe, Asia, and North Africa, and introduced into much of the rest of the world, including Australia [79,84,85]. Among the traditional medicinal practitioners in Queensland, Australia, it is valued and well known as ‘tumbling mustard’ [14]. It is listed as an environmental weed in Western Australia and Victoria. Widely naturalized in southern, central and eastern Australia, as well as in Tasmania, Norfolk Island, South Australia, and the southern and central parts of Western Australia, and many parts of the Northern Territory [76,79,84]. Seeds are used as an expectorant, a tonic for fever, used to treat bronchitis, dysentery, worms and chickenpox. Plant material is employed as a diuretic, and a decoction is used to eradicate worms and in the treatment of indolent ulcers [86].

*Sisymbrium erysimoides* Desf. commonly known as smooth mustard, or Mediterranean rocket, is a desert plant native to Middle East Arab countries and naturalized in Australia, North America and New Zealand [87,88,89]. It is found in the drier parts of southern Australia, north-west Victoria (particularly near the Murray River), New South Wales, Western Australia and the Northern Territory [78,90]. The nomadic aboriginal tribes in northwestern New South Wales and early colonists have a history of cultivation of *S. officinale*, *S. orientale* and *S. erysimoides* for a variety of food, traditional rituals and medicinal uses [91,92].

## 2. Main Active Ingredients of Mustards and Anti-Nutritional Factors

Allyl isothiocyanate, 4-hydroxybenzyl isothiocyanate and *p*-hydroxybenzyl isothiocyanate cause the sharp and hot pungency of mustards by stimulating the heat and acidity sensing TRPV ion channel, TRPV1, in the mouth and nasal cavity. Phenethyl isothiocyanate, benzyl isothiocyanate and sulforaphane are relatively less pungent. The sulfoxide group present in sulforaphane (4-methylsulfinylbutyl-ITC, CH_3_-SO-(CH_2_)_4_—**N**=**C**=**S**) is structurally similar to a thiol [(**R**–**S**-**H**) group] which produces onion or garlic-like odors in food. Due to the different composition of secondary metabolites in varieties of mustard, *B. juncea* and *B. nigra*, which are commonly used for aroma, produce more intense, robust heat, longer-lasting flavor and pungency compared to *B. alba*, which is mainly used for flavoring [13,93,94]. Temperature, toasting and addition of water change the potency and flavor due to myrosinase activation and glucosinolate degradation which also contributes to changes in the taste and flavor of the final food dish [13].

Use of mustards as food crops has some drawbacks as they contain metabolites that are considered anti-nutritional (Table 1). Research, new technology and breeding strategies have attempted to minimize the levels of anti-nutritional compounds in mustards (Table 1).

## 3. Use of Mustards as Food

In Australia and New Zealand, only canola quality mustards and rapeseeds are cultivated for the production of edible oil for human consumption. Food standards Australia and New Zealand (FSANZ), have approved the use of canola oil as safe for the Australian and New Zealand consumers due to the low levels of erucic acid that it contains as a result of directed breeding programs. At high concentrations, erucic acid can have toxic effects on the heart [109]. Other oils with high erucic acid, including certain rapeseed oils and mustard seed oils, are not commonly used as edible oils. Canola oil is also used in the form of margarine and edible blends for spreads, and the residual meal is utilized for animal feed [107]. Apart from the edible oil, the leaves and seeds are also consumed in various food forms in different parts of the world. With the migration of people from these parts to Australia and New Zealand, mustard foods are becoming popular among Australian and New Zealand people (Table 2).

In addition to being utilized directly as food, mustard seeds are used to add heat and a complexity of flavor. Mustard seed consumption was found to stimulate salivary secretion in human subjects and thus acts as a digestive stimulant [5,112], this explains their use as an appetizer in Asian, and Chinese cultures. In addition, mustards stimulate the pancreatic lipase and amylase secretion in experimental rats [118]. Yellow (*B. hirta*) or white mustard (*B. alba*) are used as spreads on burgers, hotdogs and sandwiches and widely consumed in this form in Australia and New Zealand [44].

## 4. Mustard in Food Processing

As a food additive in the food processing industry mustards are used as flavoring agents [51], emulsifiers, adhesives and biodegradable surfactants [119], suspending agents, opacifiers for sauces and dressings, natural colorants, stabilizers, and as a viscosity builder for salad dressings, sauces and marinades [2,55,64,116,120,121]. 

Mustard is used to prevent microbial growth of food-spoiling bacteria and extend the shelf life of processed food due to their anti-oxidant properties. Anti-microbial components include a wide range of glucosinolates as well as proteins which have the ability to inhibit bacterial growth in foodstuffs [113,116,122]. Anti-oxidant components such as quercetin, catechin, vitamin C and E in mustard suppress the formation of hydrogen peroxides, superoxides, peroxynitrites and thus reduce the rate of food oxidation [122].

Australia exports glucosinolate extracts from mustard in the form of volatile oil of mustard (allyl isothiocyanate) to Japan, where it is used to impart flavor to the condiment wasabi [123]. Mustards have been used historically to process, season and preserve meat, poultry, game and cold meats and meat products such as minced and canned meat as well as nuggets, sausages, salamis or hamburgers [44,124]. The quality of meat and meat products deteriorates mainly due to lipolysis from the growth of microorganisms and lipid oxidation. Anti-microbial components of mustards retain the freshness of meat, preventing bacterial growth and lipid oxidation, which increases the longevity and stability of the product [121,125,126].

The oils in the mustard seeds contain amphiphilic lipids like lecithin which consist of lipophilic fatty acid chains that are attached to a hydrophilic polar group which is water soluble. This property allows the mustard paste mucilage to be used as an emulsifier which helps to suspend oils in water and prevents the separation of ingredients in salad dressings and mayonnaise; it also has the ability to absorb liquid in food, helping to keep prepared meats firm and moist during cooking [115].

## 5. Use of Mustard in Agriculture

### 5.1. Use of Mustard as Livestock Feed

Most of the mustard leaves left over following seed collection are used as fodder [53] and seed residue is utilized as the feed for the pig, poultry, dairy and other livestock and aquaculture industries [69,95,106,127]. The oil free meal obtained after the extraction of oil contains around 37.5% protein [105]. Canola meal has a high amino acid composition, high crude and digestible protein content, rich energy content and better protein efficiency ratio than other plant derived protein sources [106]. However, the arginine (Arg) content is very low in canola meal and chickens, which cannot synthesize Arg, are greatly reliant on dietary Arg. Supplementation with soybean meal which contains high Arg levels can overcome this problem [120].

### 5.2. Use of Mustard for Companion Planting or Rotational Crop

Companion planting, mixed intercropping and rotational planting all provide benefits to agriculture; including reducing pests and pathogens, combating weeds, maintaining soil biodiversity and supplying necessary nutrients, all of which increase crop yield [43,111,128,129,130,131,132,133]. *B. alba*, *B. nigra*, *B. juncea*, *B. napus* and occasionally *B. carinata* are popular rotational or intercropping species, used with rice and wheat farming in Australia and New Zealand [133,134,135]. They offer an environmental benefit to the farmer looking for a crop to cultivate in rotation with their main crop [107]. In an intercropping field experiment, rapid cycling Brassicas including *B. rapa*, *B. alba* and *B. napus* were grown in rotation with field pea and lentils. Compared to control fields grown without rotation, it was found that brassicas significantly reduced weed biomass in the experimental field [136].

Mustard plants produce certain allelochemicals that influence the germination, growth, survival, and reproduction of other plants that are grown in proximity (allelopathic effects) [52,130,131,132,137]. The compounds 8-hydroxyquinoline and (±)-catechin present in the mustard plants are powerful chelator of nutrients such as phosphorus. These chemicals are released from the roots altering nitrogen fixation in the soil, aiding accumulation of nitrogen as well as decomposition and release of ammonium nitrogen by the soil microbiota, improving nutrient acquisition [138]. Excess nitrogen is then provided to the surrounding plants to improve crop yield. Because of this, and their weed control benefits, mustards are an excellent companion or rotational crop [137]. Moreover, all mustards contain brassinosteroids, like brassinolide, which have growth-enhancing and yield-proliferating properties [139].

Volatile sulfur compounds including glucosinolates are anti-microbial and allelopathic metabolites that have the potential to prevent bacterial infection, fungal infestation, nematode invasion in the roots and insect infestation, through a process known as biofumigation [140]. Glucosinolates are repellent to most insect pests [128,129], due to their presence in the volatile oils released from Brassicaceae [137]. Plants can be used as a natural insecticide and have been shown to be effective against aphids, red spider mites and flies [141]. The insecticidal activity of mustards is similar to permethrin, a pyrethroid. This chemical substance acts on the nerve cell membranes of insects or parasites, inhibiting the sodium channel current responsible for the polarization of the nerve cell membrane. As a consequence repolarization of the membrane is delayed and ultimately the pest is paralyzed and dies [138,142]. Constituents of mustard, for instance- aliphatic glucosinolates-allyl thiocyanate, allyl isothiocyanate, allyl isocyanate, allyl cyanide; aromatic isocyanates, phenyl, benzyl, phenethyl and 2-naphthylisothiocyanate; and nitriles e.g., 3-indolyl-acetonitrile [143]; have been shown to have insecticidal activity by inhibiting insect sodium channel currents, affecting ion permeability and homeostasis [142].

Cultivation of wheat, barley, oats, rice and maize, which are the major crops of Australia and New Zealand [22,31], using intensive non-rotational farming practices, can result in problems with weeds, soil-borne diseases and pests. Crop rotation practices can overcome some of these problems. Winter canola is immune to many pests which affect other major crops and shows higher water use efficiency [144]. Incorporation of Brassicaceae mustards as companion plant or rotational crop helps to suppress the pests and weeds and maintain crop yield. These practices are commonly used in Australia and New Zealand [15,22,111,137,138].

### 5.3. Use of Mustard in Soil Amendments, Supplementation and Bioremediation

In Australia, New Zealand and other countries, mustard crop byproducts are often utilized in soil amendments and supplements [18,107]. The residual material of mustard seeds after oil extraction, called oilcakes, or more commonly mustard meal, are used to provide valuable nutrients to the soil at a low cost, with minimal phytotoxicity, making it particularly attractive for developing countries [145]. This material provides a high quality nutrient source due to the favorable C to N ratio [146,147]. In addition, the meal is abundant in nitrogen rich proteins (~20–45%), essential nutrients and trace metal ions, namely phosphorus (~1%), potassium (~1%), calcium (~1%), magnesium (~0.5%), sulfur (~0.5% to 2%), zinc (~100 mg per kg of total dry matter), manganese (~100 mg per kg of total dry matter), and copper (~10 mg per kg of total dry matter) [133,147,148]. The material is also high in polyphenols and lignins; compounds which are considered as a slow carbon (C) pool in soil dynamic models [149].

When the decomposed seeds and oilcakes are applied to the soil they increase the abundance of water-soluble fractions of phenolics and other nutrients which can be directly absorbed by the plant and help to accelerate rapid root development and overall plant health; thereby, indirectly helping to increase host resistance to nematode invasion [150,151].

The use of plants for the removal of contaminants, including heavy metals from soils or any other form of contaminated media is termed phytoextraction and/or phytoremediation [152]. In a study out of New Zealand, *B. juncea* was found to be particularly effective for elimination of copper by phytoextraction, but also demonstrated potential for additional metal uptake from soils including cadmium, nickel, lead, and zinc [153].

## 6. Use of Mustard to Treat Ailments and Disease

Mustards have been used by Australians and New Zealanders traditionally and also in holistic herbal medicine [3,67,154]. The plant material has also been used for years for the treatment of diseases and pathological conditions (Table 3).

## 7. Mustards in Biodiesel Production

Biodiesels are an environmentally friendly biofuel which can directly replace conventional petroleum diesel needs. It is produced by a relatively simple chemical reaction called transesterification from vegetable oils or animal fats. After this fairly simple chemical reaction, canola oil converts to a methyl ester which is used as biodiesel. In this reaction glycerin is produced as a bi-product which is consumed as a raw material in the soap and cosmetic industryies. The Australian Export Grains Innovation Centre (AEGIC), Commonwealth Scientific and Industrial Research Organisation (CSIRO) and Australian Oilseeds Federation (AOF) are the agencies mostly involved in biodiesel research and production in Australia. Annually, 70 to 90 per cent of the Australian canola market, worth between $600 million to $900 million, is exported to the European Union of which 70 per cent is used to produce biofuel [23,100,105,107]. Australia follows the voluntary biofuel use policy, while New Zealand has its own mandatory biofuel sales obligation [160]. Biodiesel NZ, a nearly 90 percent state-owned company is the major stakeholder of the local biodiesel industry in New Zealand [161]. Brassicaceae mustards used in biofuel production are mainly non-GM canola type because the strict regulation of the European Union does not accept GM-based products. Meal from non-GM canola is usually sold into the livestock feed industry [23]. Due to the relative profitability of biodiesel production it is more valuable than food. The cultivation of non-GM canola for the biofuel industry is currently considered as a profitable economic strategy in Western Australia [162].

## 8. Research on Brassicaceae Mustards

Due to their economic importance as a source of high quality cooking oil, protein rich meal and other value-added products, Brassicaceae mustards are the focus of significant research attention in Australia and New Zealand [24,144,160,163,164,165]. Much of which aims to increase crop yield, through adaptation to abiotic stress, including temperature, drought, and salinity, as well as to optimize the utilization of agronomic inputs (fertilizer, insecticides and trace elements) [33,123,164]. There is also research to explore the genetic origins [20,165], development of variety traits [30,166], improve quality by targeted breeding [133,140,167] and optimization of the use of bi-products [27,133,140,162] and utilizing the secondary metabolites of Brassicaceae mustards for therapeutic purposes [168].

Australia is a member of an international consortium of scientists that reported the sequencing of the *Brassica napus* genome [169], and participated in the annotation of the draft genome for other Brassica species, i.e., *Brassica rapa* [170]. Southern Cross Plant Science at Southern Cross University in Lismore, NSW, Australia, hosts two of the large public domain global web-based databases of reference genetic data for Brassica—brassica.info (http://www.brassica.info/) and CropStoreDB (http://www.cropstoredb.org/interface.html).

## 9. Summary and Outlook

Uses of Brassicaceae mustards traditionally and in agriculture in Australia and New Zealand is widespread. Their use for various purposes from edible to therapeutic purposes began with their arrival from Europe. Mustard crops play a pivotal role in the economy of Australia and New Zealand by supplying a large amount of edible oil for human consumption and processing and the oil free meal serves as a rich protein source which is currently used as animal feed and as fertilizer. 

## Figures and Tables

**Table 1:** Major anti-nutritional factors of canola and mustard oilseeds.

Anti-Nutritional Factors	Efforts to reduce adverse effects
**Tannins**—Rapeseeds and canola contain high amount of tannin ranging up to 1973 mg of catechin equivalents/100 g of hulls [95,96]; meal constitutes about 3% tannin [97].	Tannins are less water soluble and mostly present in seed hull [95]. With the advent of a number of new procedures for dehulling and application of enzymatic processing [96,98,99,100], tannin content can lowered from rapeseed and canola meal.
**Sinapine**—Rapeseed contains high amount of phenolic compounds. For example, phenolics in rapeseed flour contains about 30 times more phenolic compounds than soybean flour [98]. One of these compounds, sinapine, which accounts for about 1–1.5% of the meal [94,96,100] has been found to be responsible to produce a “fishy” smell in brown-shelled eggs when rapeseed meal is fed to chickens [95].	Sinapine is a choline ester of sinapic acid. Its characteristic fishy taint is associated with a genetic disorder of some brown layers which cannot metabolize the choline esters properly [95,101,102,103]. The genes responsible for the fishy taint have been identified and eliminated from the chicken population by screening and breeding [102,103]. Moreover, limiting the supplementation of choline in diet, can overcome the problem and diets including 10–12% canola meal are not harmful to the poultry [95,103].
**Phytic acid**—Regarded as an anti-nutritional factor, phytic acid, which is composed primarily of phosphorous and inositol, strongly binds to metallic cations (Ca, Fe, Zn, Mn, Mg), forming insoluble complexes and interfering with their absorption [95].	Recombinant DNA technology has led to the production of phytases enzyme with improved functional properties that hydrolyses phytic acid to inositol and inorganic phosphorus, resulting high phosphorus utilization and overall growth performance of monogastric animals [95].
**Nitriles**—Indoles present in the mustard seeds in varying amounts can cause a pneumonia type syndrome in goats. Nitriles have the potential to create preliminary liver damage with secondary photosensitization, and/or brain damage characterized by loss of sight. Build up of nitrates can also cause respiratory problems and death [104].	Before feeding, levels of secondary metabolites including erucic acid and glucosinolates should be checked and selection of meal containing low levels will avoid toxicities [101,105,106].
**Isothiocyanates**—Another group of plant secondary metabolite, isothiocyanates can produce digestive disturbances in goats involving rumen stasis and constipation. Non-protein amino acids present in the seed meal can create anemia with blood colored urine [104].	Selection of meal containing low levels of isothiocyanates will avoid the deleterious effects [104,107,108].

**Table 2:** Uses of mustard as a main food ingredient.

Common Use	Mustards	References
Edible oil	All mustards mentioned here produce seeds which contain significant amounts of oil. Mustard seed oil is popular in Indian cooking and often used as a substitute for ghee, an Asian form of clarified butter.	[50,108,110]
Condiment	Seeds of *B. alba*, *Neslia paniculata*, *B. nigra*, *B. napus*, and *B. juncea* are widely used as a condiment to impart a hot pungent flavor to food, either alone or with other spices. The seeds of *B. nigra* have the strongest intensity because of the high glucosinolate content. Raw whole mustard seeds, toasted, and ground as paste are used in hundreds of curries, snacks, sauces and recipes for the addition of heat and a depth of flavor.	[49,50,51,110,111,112,113]
Vegetable	*Brassica* mustard species are cooked similar to spinach. They are also fermented and used as popular mild flavored leafy vegetables, Kimchi, a popular traditional Korean dish, which is popular in Australia is made from fermented *B. juncea*, *B. nigra* and *S. officinale.*	[50,110]
Salad	Leaves from *B. nigra*, *B. alba*, *B. juncea*, *B. napus,* and *C. irregularis* are consumed as salad greens.	[49,56,113,114]
Sauce	Mustards are used to enhance the piquancy and texture of several types of sauces, and are important ingredients of English mustard, Dijon mustard, vinaigrettes and Chinese hot mustard.	[115]
Artificial wasabi	Powdered mustard is blended with dried horseradish and green dye to produce wasabi paste.	[116]
Fodder	Both the leaves and seed residues of *B. napus*, and *Neslia paniculata* are used as fodder for both monogastric and ruminant livestock.	[117]

**Table 3:** Traditional/folkloric use of mustard extracts.

Traditional Use	Mustards	References
Anti-microbial activity	*Alliaria petiolata, Sisymbrium officinale, S. erysimoides, Brassica hirta*, *B. nigra*	[44,45,110,121,126,155]
Anti-diabetic activity	*B. juncea, B. nigra*	[7,110]
Treatment in vitamin C deficiency	*A. petiolata, B. rapa, B. napus*	[110]
Diuretic activity	*A. petiolata*, *B. juncea*, *B. napus*, *B. nigra*, *B. rapa*, *S. officinale*, *S. orientale*	[80,81,156]
Expectorant activity	*A. petiolata*, *S. orientale*, *S. officinale*, *S. erysimoides*	[80,81,156]
Stimulant activity	*A. petiolata*, *B. alba*, *B. juncea*, *B. nigra*	[157]
Analgesic activity	*B. carinata*, *B. juncea*, *B. napus*, *B. rapa*, *Calepina irregularis, Neslia paniculata,* *S. erysimoides*, *S. officinale*	[110]
Activity in cold and flu	*B. alba*, *S. officinale*, *S. erysimoides*, *B. napus*, *B. nigra*	[44,156]
Anti-catarrhal activity	*B. alba*, *S. officinale*, *S. erysimoides*, *B. napus*, *B. nigra*	[44,156]
Bronchitis	*S. officinale*, *S. orientale*, *S. erysimoide*s.	[86]
Anti-asthmatic activity	*S. officinale*	[80,81]
Emetic activity	*B. alba*, *B. nigra*, *B. juncea, S. officinale*, *B. nigra*	[110]
Anti-cancer activity	*B. juncea*, *B. napus*, *B. rapa*, *S. officinale*	[110]
Laxative	*B. alba*, *B. nigra*, *B. juncea, S. officinale*	[110]
Rubefacient	*B. juncea*, *S. officinale,* *B. rapa*, *B. juncea*	[80,81]
Galactagogue	*B. juncea*	[110]
Anti-gout potential	*B. napus*, *B. rapa*	[64,110,158]
Use in gall stone	*B. napus*, *B. rapa*	[44,54,66,84]
Use against alopecia	*B. nigra*	[159]
Anti-dandruff activity	*B. nigra*	[44,67]
Use in neuralgia	*B. nigra*	[44,67]
Anti-spasmodic activity	*B. nigra, S. officinale*.	[44,67,84]
Aphrodisiac activity	*B. rapa*, *B. nigra*	[44,53,67]
Use in hepatic and kidney colic	*B. rapa*	[44,53,84]
Anti-inflammatory activity	*B. rapa*, *S. erysimoides, S. officinale*	[44,53,84]
Anthelmintic activity	*B. rapa,* *S. orientale*	[44,53,66,84]
Remedial use in fever	*S. orientale*	[76,79,86]
Use in dysentery	*S. orientale*	[76,79,86]
Anti- addiction activity	*S. officinale*	[83]
Appetizing, digestive and aperitif activity	*S. officinale*, *B. nigra*, *B. juncea*	[44,79,83]
Snake bite antidote	*B. rapa*, *S. officinale*	[14,44,52,78,82,85]
Skin disorders	*Neslia paniculata*	[4]
